# Monitoring the Risk of Type-2 Circulating Vaccine-Derived Poliovirus Emergence During Roll-Out of Type-2 Novel Oral Polio Vaccine

**DOI:** 10.3390/vaccines12121308

**Published:** 2024-11-22

**Authors:** Corey M. Peak, Hil Lyons, Arend Voorman, Elizabeth J. Gray, Laura V. Cooper, Isobel M. Blake, Kaija M. Hawes, Ananda S. Bandyopadhyay

**Affiliations:** 1 Bill & Melinda Gates Foundation, Seattle, WA 98109, USA; hil.lyons@gatesfoundation.org (H.L.); arend.voorman@gatesfoundation.org (A.V.); kaija.hawes@gatesfoundation.org (K.M.H.); ananda.bandyopadhyay@gatesfoundation.org (A.S.B.); 2 MRC Centre for Global Infectious Disease Analysis, School of Public Health, Imperial College London, London W12 0BZ, UK; e.gray@imperial.ac.uk (E.J.G.); l.cooper@imperial.ac.uk (L.V.C.); isobel.blake@imperial.ac.uk (I.M.B.)

**Keywords:** polio, novel oral polio vaccine, circulating vaccine-derived poliovirus, emergence risk

## Abstract

**Background/Objectives:** Although wild poliovirus type 2 has been eradicated, the prolonged transmission of the live- attenuated virus contained in the type-2 oral polio vaccine (OPV2) in under-immunized populations has led to the emergence of circulating vaccine-derived poliovirus type 2 (cVDPV2). The novel OPV2 (nOPV2) was designed to be more genetically stable and reduce the chance of cVDPV2 emergence while retaining comparable immunogenicity to the Sabin monovalent OPV2 (mOPV2). This study aimed to estimate the relative reduction in the emergence risk due to the use of nOPV2 instead of mOPV2. **Methods:** Data on OPV2 vaccination campaigns from May 2016 to 1 August 2024 were analyzed to estimate type-2 OPV-induced immunity in children under 5 years of age. Poliovirus surveillance data were used to estimate seeding dates and classify cVDPV2 emergences as mOPV2- or nOPV2-derived. The expected number of emergences if mOPV2 was used instead of nOPV2 was estimated, accounting for the timing and volume of nOPV2 doses, the known risk factors for emergence from mOPV2, and censoring due to the incomplete observation period for more recent nOPV2 doses. **Results:** As of 1 August 2024, over 98% of the approximately 1.19 billion nOPV2 doses administered globally were in Africa. We estimate that approximately 76 (95% confidence interval 69–85) index isolates of cVDPV2 emergences would be expected to be detected by 1 August 2024 if mOPV2 had been used instead of nOPV2 in Africa. The 18 observed nOPV2-derived emergences represent a 76% (74–79%) lower risk of emergence by nOPV2 than mOPV2 in Africa. The crude global analysis produced similar results. Key limitations include the incomplete understanding of the drivers of heterogeneity in emergence risk across geographies and variance in the per-dose risk of emergence may be incompletely captured using known risk factors. **Conclusions:** These results are consistent with the accumulating clinical and field evidence showing the enhanced genetic stability of nOPV2 relative to mOPV2, and this approach has been implemented in near-real time to contextualize new findings during the roll-out of this new vaccine. While nOPV2 has resulted in new emergences of cVDPV2, the number of cVDPV2 emergences is estimated to be approximately four-fold lower than if mOPV2 had been used instead.

## 1. Introduction

The oral polio vaccine (OPV), developed by Albert Sabin and used on a mass scale beginning in 1960 [1], is an essential tool for the Global Polio Eradication Initiative (GPEI) and has been remarkably successful at providing both individual and population-level protection against poliovirus infection and poliomyelitis [2]. The vaccine contains a live-attenuated virus that can produce a strong mucosal and humoral immune response following replication in the intestines and, once shed in stool, can yield secondary protection to contacts of the vaccine recipient. However, the prolonged transmission of the vaccine virus in under-immunized populations can lead to the loss of attenuation and emergence of circulating vaccine-derived poliovirus (cVDPV) [3].

Although wild poliovirus type 2 has been eradicated, cVDPV type 2 (cVDPV2) continues to spread and presents a catch-22 challenge to poliovirus eradication as the Sabin monovalent OPV type 2 (mOPV2) and trivalent OPV (tOPV) used to stop cVDPV2 outbreaks have also seeded emergent cVDPV2 outbreaks [4,5]. This challenge has grown dramatically following the April 2016 “Switch” from trivalent to bivalent OPV, which removed the type 2 component from routine OPV immunization [6]. A 2023 analysis of post-Switch cVDPV2 outbreaks in the African continent quantified the association between factors such as lower immunity and larger campaign size with an increasing risk of cVDPV2 emergence [7].

The novel OPV2 (nOPV2) was designed in part to be more genetically stable and reduce the chance of cVDPV2 emergence while retaining comparable immunogenicity to mOPV2 [8]. Among other design features, nOPV2 includes a stabilized domain V region [9]. In mOPV2, domain V is a key attenuation site that rapidly reverts to the wild type within an estimated mean time of 6.5 days [10]. Pre-clinical and clinical data on nOPV2 have shown that the vaccine is safe and immunogenic and has substantially lower rates of reversion and neurovirulence compared to mOPV2 [11,12].

Based on pre-clinical and clinical trial data and the designation of polio as a Public Health Emergence of International Concern [13], nOPV2 received the first-ever Emergency Use Listing (EUL) by the World Health Organization (WHO) [14] and its use rapidly scaled up to millions of recipients through -outbreak responses under close field monitoring and enhanced surveillance to ensure the safety, effectiveness, and genetic stability of nOPV2 [15]. Viruses observed in the field can be distinguished as originating from nOPV2 or Sabin-based mOPV2 or tOPV based on evidence from whole genome sequencing including the presence of any of the nOPV2 design features [16].

As of 1 August 2024, 18 cVDPV2 emergences linked to nOPV2 had been reported, all from the African continent [17]. This study aimed to estimate the relative reduction in emergence risk from the use of nOPV2 instead of mOPV2. In this paper, we compare cVDPV2 emergence events observed following nOPV2 use to the number of emergence events that could have been expected if mOPV2 were used instead.

## 2. Materials and Methods

### 2.1. Vaccination Campaigns

Since the ‘Switch’ from trivalent OPV (tOPV; types 1, 2, and 3) to bivalent OPV (bOPV; types 1 and 3) in April 2016, type-2 OPV has only been used in supplemental immunization activities (SIAs). Data from each SIA marked as completed and with a start date from May 2016 through July 2024 were downloaded on 1 August 2024 from the Polio Information System (POLIS) database maintained by the WHO [18]. Data access was provided through the Global Polio Eradication Initiative Data Sharing Agreement. As this study was conducted retrospectively using routinely collected anonymized data on vaccinations and poliovirus detections, informed consent was not required or obtained. The number of OPV doses was estimated based on target population by both campaign name as well as by province and manually cross-checked against offline vaccination tracking data for recent campaigns. Total province population size was estimated assuming that the under-5 SIA target population size represented 17% of the population demographic.

### 2.2. Immunity Estimation

Type-2 OPV-induced immunity for children aged under 5 years was estimated for each district and month using a dynamic model assuming 80% per-campaign coverage [19]. To estimate the pre-campaign immunity in each province, we calculated the population-weighted average district immunity for the month prior to each campaign start date.

### 2.3. Virus Surveillance

Poliovirus surveillance depends on isolation of the virus from human stool specimens (from acute flaccid paralysis surveillance or contact sampling) or sewage samples from environmental surveillance (ES) [20]. Standard guidelines include up to 10 days in cell culture followed by Sanger sequencing. Data on polioviruses were downloaded from POLIS and cVDPV2 emergence groups were assigned as derived from nOPV2 or Sabin-based mOPV2 or tOPV based on classification by whole genome sequencing [16]. Provinces were classified as having ES if there was at least one ES result in the past year.

### 2.4. Estimating Seeding Date

The mean seeding date for cVDPV2 was estimated based on the number of nucleotide changes in the VP1 region of the index isolate compared with mOPV2, using a mutation clock estimated as two instantaneous mutations and then nine changes per year following a Poisson process [10]. cVDPV2 emergence groups with index viruses with mean estimated seeding date after April 2016 were counted as emergences seeded after the Switch. All analyses were performed in R (version 4.1.2; R Core Team).

### 2.5. Estimating Time to Detection

To account for the time lag between the use of OPV2 and the detection of any subsequent emergences, we estimated the distribution of emergence waiting times and reporting waiting times (surveillance lags). The emergence waiting time distribution is defined as the time from the potential seeding event (i.e., the OPV2 SIA) to the index virus date and was estimated using a mixture model of mutations given OPV2 exposures in SIAs and surveillance type. The viral age of the index isolate in each emergence group was subtracted from the virus date (i.e., AFP onset date or ES collection date) to generate a probabilistic time period during which seeding may have occurred. OPV2 exposure was estimated using the SIA starting date, number of OPV2 doses, and distance to district of emergence based on a radiation model of decay over space [19]. Emergence waiting time was estimated with a log-normal distribution for all emergences and again excluding ES to reflect waiting times in a setting with only AFP surveillance.

The reporting waiting time is defined as the time from virus date (i.e., AFP onset or ES collection date) until notification of VP1 viral sequence. Focusing on cVDPV2 detections after 1 May 2016, we estimated the waiting time using a mixture of gamma distributions with random effects per country for the mean and shape parameters. We ignored isolates that took longer than 1 year to report, which were likely de-prioritized samples as compared with samples of potential epidemiological importance such as potential new emergences. Since the sequencing lag began after the emergence waiting time, the convolution of the distributions was calculated to construct the time to detection of the index isolate for a new emergence group.

### 2.6. Estimating Number of Emergences Expected

We first performed a global crude analysis based on the observed emergence rate per post-Switch mOPV2 or tOPV dose. Scaling this rate by the number of nOPV2 doses used provided a total number of emergences that would have been expected if each nOPV2 SIA during the period from March 2021 through July 2024 had been replaced with mOPV2. The Poisson distribution was used to construct 95% confidence intervals. The time to detection distribution following each nOPV2 SIA was then scaled such that the area under the curve equaled the expected number of emergences from that SIA. The expected number of emergences detected by a given day could then be estimated as the cumulative sum up to that day.

Given that the vast majority of nOPV2 doses had been administered in the African continent, and previous analyses had estimated the relationship between campaign size, pre-existing immunity, and the consequent cVDPV2 emergence rate from mOPV2, we next performed an adjusted analysis focused on Africa. Using the framework and fitted parameters $\theta_{size}$ and $\theta_{u5}$ reported by Gray et al. [7], we estimated $E[Y_{n}]=\hat{\alpha }\sum_{i\in C_{n}}exp\left(\left(\theta_{size}\right)\log\left(x_{size,i}\right)+\theta_{u5}x_{u5,i}\right)$, where $E[Y_{n}]$ is the expected number of emergences from a set $C_{n}$ of EUL-period nOPV2 SIAs, each with size $x_{size,i}$ and pre-campaign immunity $x_{u5,i}$. The scaling factor $\hat{\alpha }$ is estimated based on the $Y_{s}$ observed emergences from a set $C_{s}$ of post-Switch mOPV2 or tOPV SIAs according to $\hat{\alpha }=\frac{Y_{s}}{\sum_{i\in C_{s}}exp\left(\left(\theta_{size}\right)\log\left(x_{size,i}\right)+\theta_{u5}x_{u5,i}\right)}$. To account for parameter uncertainty, $E[Y_{n}]$ was calculated for each of 500 samples from the joint posterior ${(\theta_{size},\;\theta }_{u5})$ Markov Chain Monte Carlo output. The 95% prediction interval was constructed using the 2.5th and 97.5th percentiles. As per the crude analysis, the number of expected emergences was distributed based on the time to detection.

## 3. Results

### 3.1. Description of Post-Switch OPV2 Use

**Figure 1:**
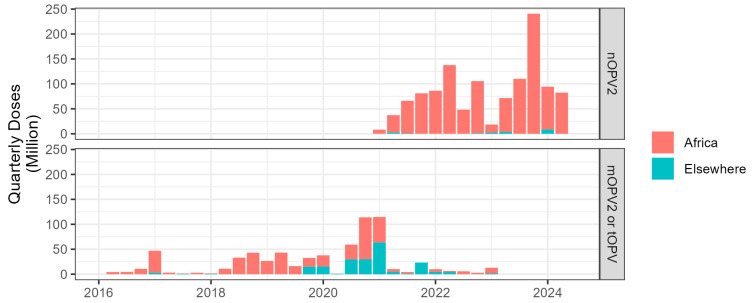
Quarterly number of OPV2 doses by vaccine product.

Between 1 May 2016 and 1 August 2024, 127 million doses of tOPV, 551 million doses of mOPV2, and 1.19 billion doses of nOPV2 were administered (Table 1). The use of nOPV2 increased rapidly since its first use in March 2021 and represented 100% of total OPV2 use since Q2 2023 (Figure 1). While global crude results are presented, given that 1.17 of the 1.19 billion nOPV2 doses (98%) used were in Africa, the main results in this paper focus on emergence risk in Africa, with spotlights on Nigeria, the Democratic Republic of the Congo (DRC), and other priority countries.

**Figure 2:**
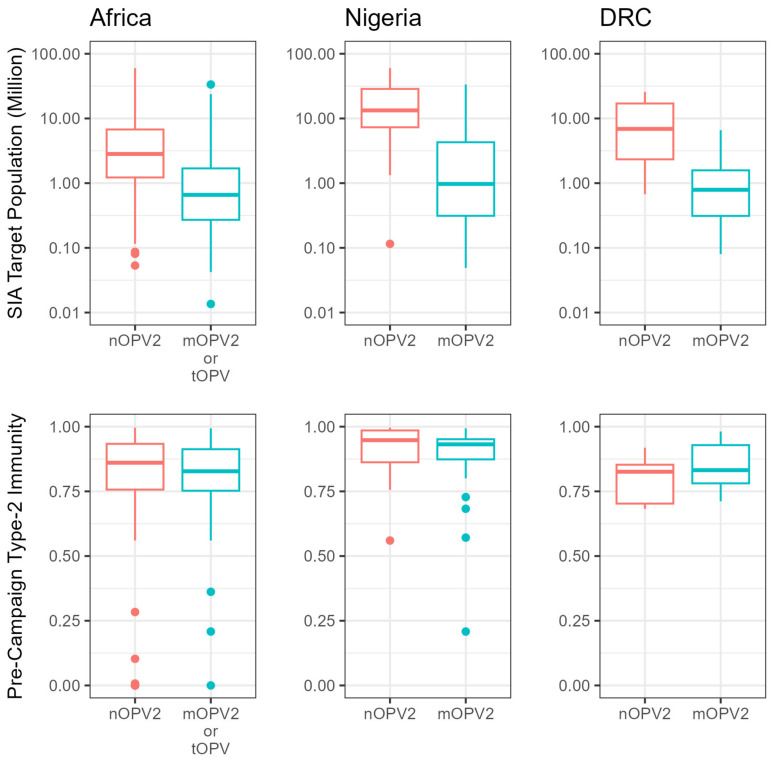
Boxplots of SIA size (**top**, note log axis) and pre-campaign immunity (**bottom**) for Africa, Nigeria, and DRC. Note that no tOPV was used in Nigeria or DRC during the period from 1 May 2016 to 1 August 2024.

The median target population size was smaller for post-Switch campaigns in Africa with mOPV2 or tOPV (646,204 [IQR 266,388–1,690,858]) than nOPV2 (2,766,913 [IQR 1,195,384–6,159,151]) (*p* < 0.001) (Figure 2). The estimated pre-campaign type-2 mucosal immunity among children aged under 5 years was similar for post-Switch campaigns in Africa with mOPV2 or tOPV (median 0.83 [IQR 0.75–0.91]) and nOPV2 (median 0.86 [IQR 0.76–0.93]) (*p* = 0.15) (Figure 2), though more nOPV2 was generally used in lower-immunity contexts than mOPV2 in the DRC.

### 3.2. Observation of Post-Switch cVDPV2 Emergences

A total of 87 cVDPV2 emergences derived from mOPV2 or tOPV have been reported since 1 May 2016, of which 80 had an estimated seeding date after the “Switch” in April 2016. A total of 59 of these emergences were first detected in Africa (Table 1). The crude observed seeding risk per 100 million mOPV2 or tOPV doses was 12.2 in Africa and 10.7 elsewhere.

**Figure 3:**
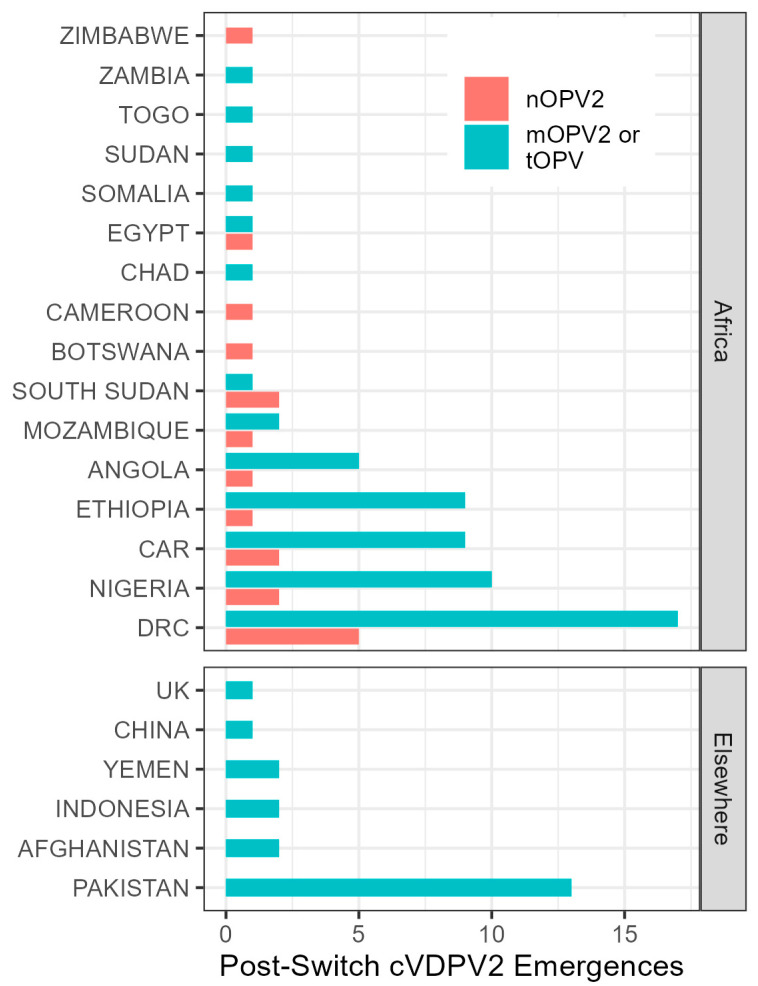
Countries detecting index isolate of cVDPV2 emergences seeded after April 2016.

A total of 18 cVDPV2 emergences derived from nOPV2 have been reported, all from Africa (Figure 3). The crude observed seeding risk per 100 million nOPV2 doses is 1.5 in Africa; however, the process for emergence and detection by surveillance creates a time lag such that emergences due to more recent campaigns (largely nOPV2) are only partially observed.

### 3.3. Estimating Emergence Expectation for nOPV2

**Figure A1:**
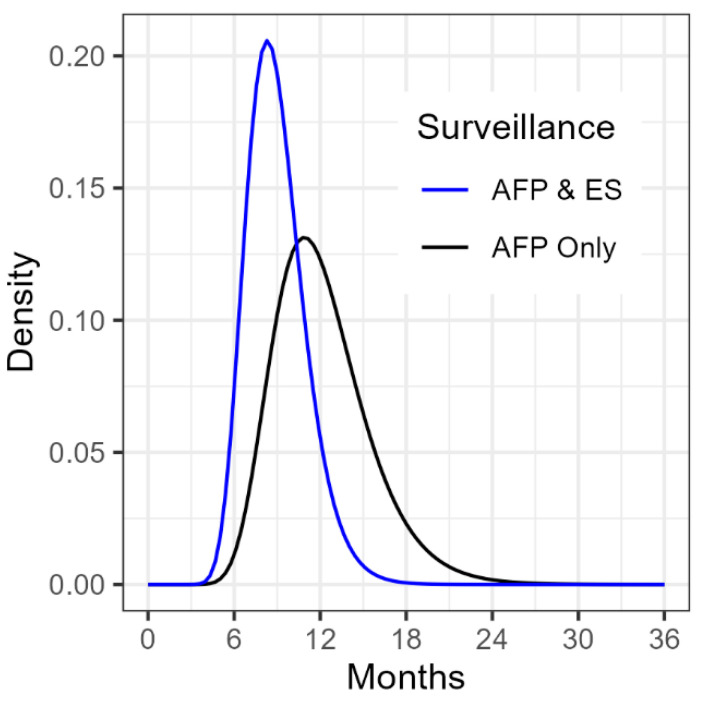
Emergence waiting time distributions.

**Figure A2:**
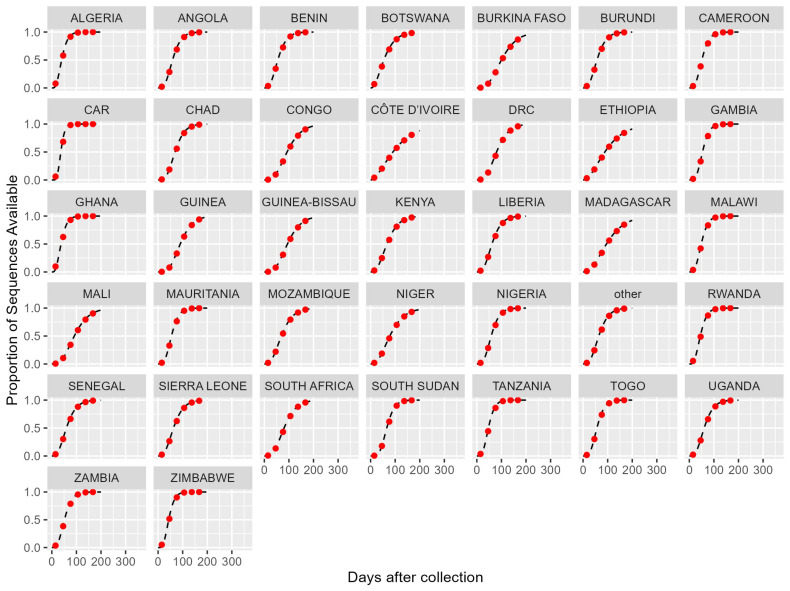
Reporting waiting time distributions. “Other” indicates other countries globally. Red dots indicate monthly mean values at monthly midpoints. Central African Replublic (CAR) and Democratic Republic of the Congo (DRC) abbreviated for fit.

Accounting for both the estimated viral age as well as the timing and proximity of SIAs, the onset date for the index virus of an estimated 60% of cVDPV2 emergences was detected by AFP surveillance within 12 months of the likely SIA seeding event; where there was AFP and environmental surveillance, 94% of index viruses were collected within 12 months of the SIA (Appendix A, Figure A1). The time from the virus date until the reporting of the viral sequence was estimated for each country; for example, 50% of sequences were reported within 56 days of the virus date in Nigeria and 77 days in the Democratic Republic of the Congo (Appendix A, Figure A2).

### 3.4. Crude Global Analysis

A crude global emergence rate of 11.8 cVDPV2 emergences per 100 million mOPV2 or tOPV doses was estimated based on 80 cVDPV2 emergences derived from 679 million post-Switch mOPV2 or tOPV doses. Applying this crude rate to the 1.19 billion nOPV2 doses used, an estimated 140 (95% confidence interval 117–164) cVDPV2 emergences would have been expected if mOPV2 had been used instead of nOPV2. However, not all of these emergences would have been observable by August 2024. Applying the estimated emergence waiting time and reporting waiting time distributions, the index isolate for 93 (75–113) of these emergences would have been expected to have been discovered by 1 August 2024. The 18 cVDPV2 emergences from nOPV2 that had been discovered by then represented an estimated 81% (76–84%) lower risk of emergence by nOPV2 as compared to mOPV2.

### 3.5. Adjusted Africa Analysis

**Figure 4:**
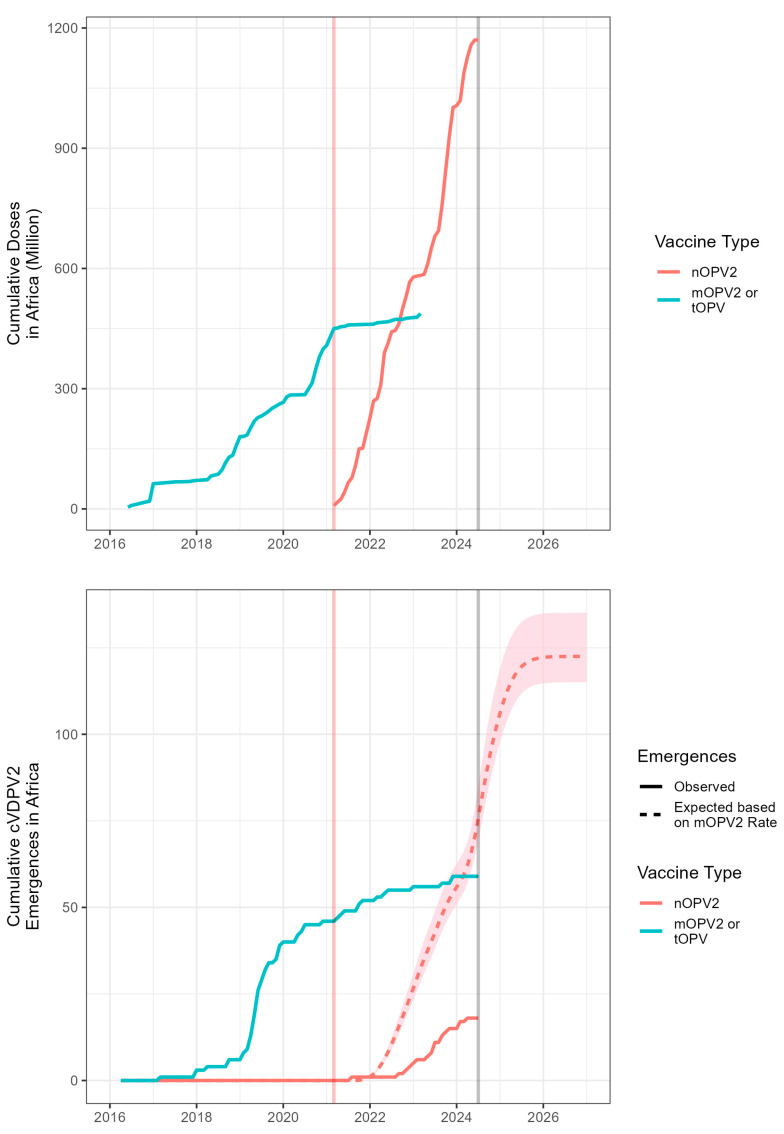
(**Top**) Cumulative doses of nOPV2 and mOPV2 or tOPV in Africa; (**bottom**) cumulative observed and expected cVDPV2 emergences derived from nOPV2 and mOPV2 or tOPV in Africa by virus date of index isolate. Vertical lines indicate date of first nOPV2 use (red) and August 2024 (grey).

An adjusted analysis was performed for the African continent, where the vast majority of nOPV2 doses have been used and where additional analyses on emergence risk factors have been performed [7]. An estimated 123 (115–135) cVDPV2 emergences would have been expected if mOPV2 had been used instead of nOPV2 given the total nOPV2 use in Africa during the period from March 2021 to 1 August 2024. Applying the estimated waiting time and reporting time distributions, the index isolate for 76 (69–85) of these emergences would have been expected to have been discovered by 1 August 2024 (Figure 4). The 18 cVDPV2 emergences from nOPV2 that had been observed by then represent an estimated 76% (74–79%) lower risk of emergence by nOPV2 as compared to mOPV2 in Africa.

### 3.6. Sensitivity Analyses

**Figure A3:**
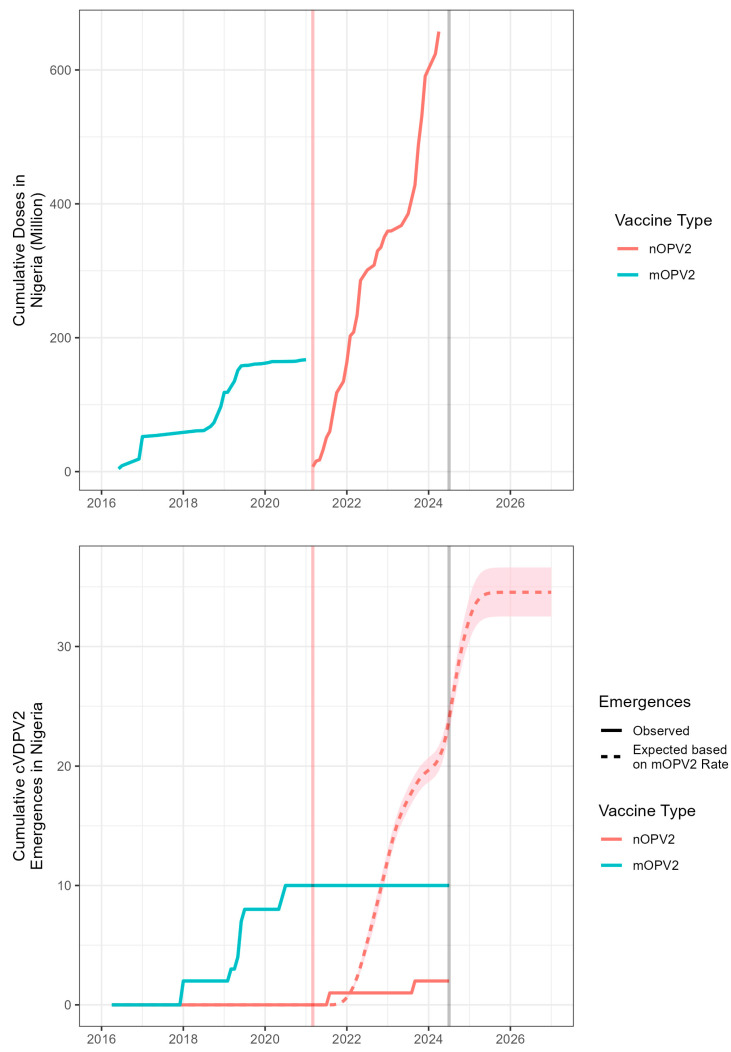
(**Top**) Cumulative doses of nOPV2 and mOPV2 in Nigeria; (**bottom**) Cumulative observed and expected cVDPV2 emergences derived from nOPV2 and mOPV2 in Nigeria by virus date of index isolate. Vertical lines indicate date of first nOPV2 use (red) and April 2024 (grey). Note that no tOPV was used in Nigeria during this period.

**Figure A4:**
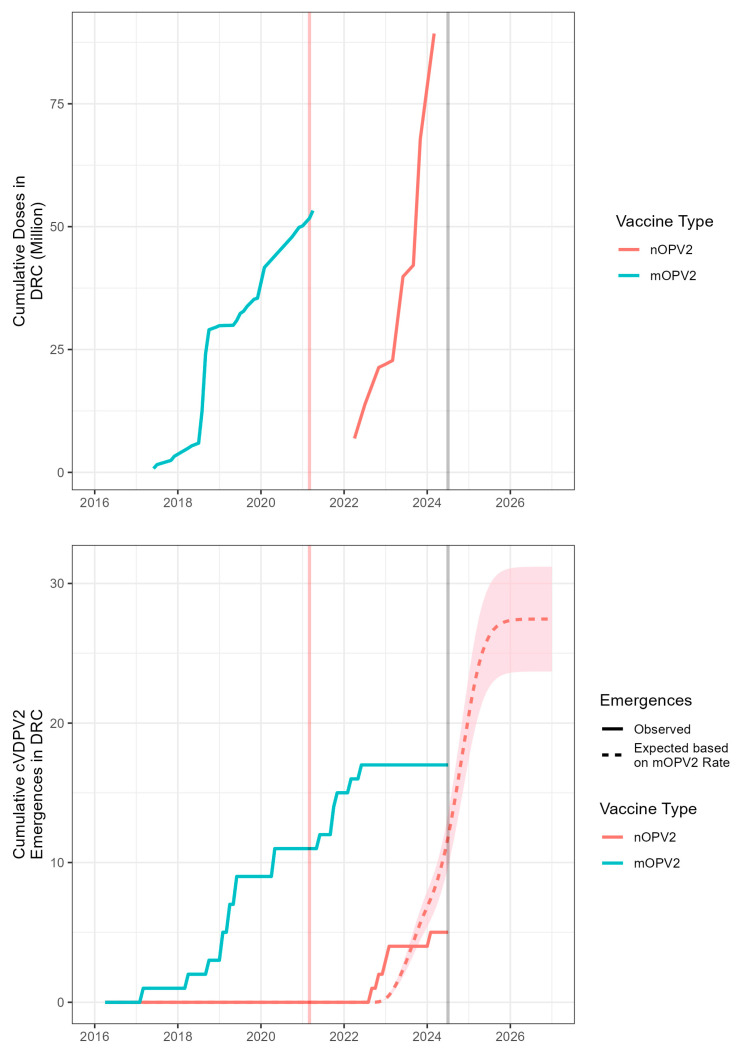
(**Top**) Cumulative doses of nOPV2 and mOPV2 in DRC; (**bottom**) Cumulative observed and expected cVDPV2 emergences derived from nOPV2 and mOPV2 in DRC by virus date of index isolate. Vertical lines indicate date of first nOPV2 use (red) and April 2024 (grey). Note that no tOPV was used in DRC during this period.

There remains residual uncertainty in estimatingthe emergence risk by location after considering known risk factors [7]. The main results narrow the scope to Africa, representing 98% of nOPV2 use, though sub-regional geographic heterogeneity in emergence risk remains. In Nigeria, 10 cVDPV2 emergences derived from 163 million doses of mOPV2 and 2 cVDPV2 emergences from 657 million doses of nOPV2 have been observed (Table 1). Estimating emergence expectations for Nigeria separately, approximately 24 (23–25) cVDPV2 emergences could have been expected by 1 August 2024 if mOPV2 were used instead of nOPV2, representing an estimated 92% (91–92%) lower risk of emergence by nOPV2 as compared to mOPV2 (Appendix A, Figure A3). Conversely, estimating emergence expectations for the DRC separately, the observed five cVDPV2 emergences derived from nOPV2 represented a 57% (48–63%) decrease compared with the twelve (10–13) cVDPV2 emergences that could have been expected by 1 August 2024 if mOPV2 were used instead (Appendix A, Figure A4).

While fifty-nine cVDPV2 emergences derived from mOPV2 or tOPV have been observed since 1 May 2016 in Africa, a cluster of four emergences from April to June 2019 in two provinces in Angola have been hypothesized to have related origins, and likewise for a cluster of five emergences in May 2019 from two provinces in the Central African Republic [21]. By treating the Angolan cluster as a single emergence event, and likewise for the Central African Republic cluster, we can repeat the analysis based on a lower emergence risk for mOPV2 and estimate that 67 (61–75) cVDPV2 emergences could have been expected by 1 August 2024 if mOPV2 had been used instead of nOPV2 in Africa, representing a 73% (71–76%) lower risk of emergence by nOPV2 as compared to mOPV2. However, consolidating these clusters is expected to influence the parameters of the emergence risk analysis in a way not accounted for here.

## 4. Discussion

These results support the growing body of evidence from clinical and field studies demonstrating that nOPV2 is more genetically stable and less likely to revert and lead to cVDPV2 emergence than mOPV2. The risk of cVDPV2 emergence is estimated to be four-fold lower following nOPV2 use than mOPV2 use. Importantly, the risk of cVDPV2 emergence can be further reduced by implementing high-quality campaigns to prevent the circulation, and hence reversion, of all types of polioviruses. These results support the use of nOPV2 for responses to cVDPV2 outbreaks and as a critical tool on the path to polio eradication. Previous work [22] provided preliminary estimates of a ten-fold reduction in emergence risk for nOPV2 compared with mOPV2 based on a smaller, earlier set of data and before accounting for the times to cVDPV2 emergence and reporting, which have been included here to address the partially observed nature of the real-time monitoring of emergence risk.

The encouraging field performance of nOPV2 underscores the importance of the tool for containing the spread of current outbreaks while carrying a lower risk of seeding new emergences. Risk assessments for scoping outbreak response campaigns must still be mindful of emergence risk [23,24], though the lower emergence risk with nOPV2 than mOPV2 shifts the balance towards larger campaigns to stop spread. However, the seeding risk is not zero, underscoring the importance of other means to reduce the emergence risk, including implementing high-coverage SIAs in rapid succession [24,25].

This work is subject to limitations including the following. First, the risk of cVDPV2 emergence is heterogeneous and its drivers are incompletely understood and modeled. For example, further study is warranted to characterize the role of non-polio enteroviruses (NPEVs) as recombination partners on the critical pathway to cVDPV2 emergence and the spatiotemporal prevalence of these NPEV species [11,26]. Immunity estimation may only partially address variation in the emergence risk across repeated SIAs in the same location; due to the large volume of nOPV2 used in Nigeria beyond the second round, emergence risk may attenuate more, or less, quickly than immunity estimate capture alone. Further, the model does not capture differences in emergence risk by relative campaign frequency. For example, all four nOPV2-derived emergences in the DRC appeared consistent with seeding during the first nOPV2 round in a province that had no OPV2 use within 2–4 years, and which was followed by a second round approximately 12 weeks later, thereby allowing time for nOPV2 viruses to circulate and revert. Reviewing such evidence, the WHO SAGE in 2023 reiterated the recommendation to plan for a second SIA no later than 4 weeks after the first SIA in a location [25].

Second, the emergence waiting time distribution has been fitted to data on cVDPV2 emergences derived from mOPV2 or tOPV, and comparisons to this waiting time assume that the waiting time for nOPV2 would be similar. We posit that this is a conservative assumption since the biological pathway to cVDPV2 emergence via recombination (the predominant reversion pathway for nOPV2) is expected to occur more quickly than the pathway via accumulation of point mutations (plausibly the predominant reversion pathway for mOPV2 [10]). We also posit that the Poisson process for estimating viral age is robust to the biological pathway by allowing for two instantaneous mutations (accounting for Sabin type 2 sites under strong selection such as A481G [10]) followed by an average of nine sites per year (such as synonymous VP1 substitutions, which would accumulate with or without recombination outside VP1). Furthermore, changes in surveillance sensitivity with time, especially increases in ES [27], may have resulted in faster surveillance more recently. Therefore, if any cVDPV2 emergences occur at all, one may expect to see them more quickly following campaigns with nOPV2 than mOPV2, and therefore, the mOPV2 comparator in this analysis may provide a conservative safety margin in this respect. Furthermore, the definition of a cVDPV2 emergence group requires an index isolate and linked confirmatory isolate. However, the median time from the index to the confirmatory isolate for post-Switch cVDPV2 emergence groups in Africa is approximately 27 days, suggesting that this introduces a lag of approximately one month.

Third, the mOPV2 comparator assumes that an equal number of mOPV2 doses would have been used instead of nOPV2, and, therefore, that no additional doses of nOPV2 are needed for a given response compared with mOPV2. The growing evidence base supports the similar immunogenicity, efficacy, and field effectiveness values of nOPV2 and mOPV2 [17,28,29,30].

Fourth, the non-differential measurement error can introduce noise for several data fields. The number of doses used in each campaign is estimated based on the target population for the SIA, the quality of which varies by location. Due to limited surveillance sensitivity, including the low case-to-infection ratio for polio [31], the location where an emergence is first detected may not be the location where the virus was first circulating and reverted. This introduces noise into understanding the risk factors for where emergence occurs and not just where emergence is first detected (which may be a function of higher relative surveillance sensitivity or transmission proclivity). Districts with an ES site were assumed to follow the ES + AFP time to discovery distributions, which did not account for potential differences in site sensitivity. The estimation of immunity depends on an assumed 80% per-round vaccination coverage with random missingness while in reality, sub-district pockets of lower coverage are expected to be important drivers of transmission and potentially emergence. In the case studies of Nigeria and the DRC, the quality of each campaign is uncertain, with potentially meaningful implications on the differences observed in these countries.

The strengths of this study include the inclusion of data monitoring over one billion doses of nOPV2, allowing for sufficient power to detect the rare event of cVDPV2 emergence. This method leverages information on campaign sizes, population immunity, and known emergence risk factors and considers lags in disease emergence and detection processes, allowing for real-time monitoring for the relative risk of emergence due to the novel vaccine compared with mOPV2. This results from this method were reviewed regularly throughout the EUL period and were critical for the WHO, SAGE, and the independent Global Advisory for Vaccine Safety (GACVS) oversight of the roll-out of nOPV2 [32].

Live-attenuated vaccines for diseases other than polio, such as rotavirus [33] and measles [34,35], have limited reports of vaccine-associated infection. While the public health impact of community spread presents a unique challenge of cVDPV, this framework, which considers both the times of vaccine use (i.e., SIA dates) as well as timelines for the development (e.g., mutation and/or recombination) and detection (i.e., surveillance) of AEFIs, may be useful for vaccines other than OPV. The log-normal and gamma functional forms used for statistical fitting provide flexibility to fit a wide range of data due to differences in surveillance or pathogen natural history. This study addressed the specific challenge of monitoring the risk of cVDPV, extending beyond the standard approaches of observed-to-expected analyses performed in near-real time for AEFIs such as for COVID-19 vaccines [36,37].

Further work to understand the risk factors for emergence would help inform this analysis and support the generalization of novel OPV types 1 and 3 under development. Furthermore, while a lower emergence risk is unequivocally better, a key question remains—how low emergence risk must be to achieve eradication under various programmatic implementation conditions.

## 5. Conclusions

While nOPV2 has resulted in new emergences of cVDPV2, the number of cVDPV2 emergences is estimated to be approximately four-fold lower than if mOPV2 had been used instead. These results are consistent with the accumulating clinical and field evidence showing the enhanced genetic stability of nOPV2 relative to mOPV2. By accounting for detection timelines, this approach has been and can continue to be updated in near-real time to contextualize new findings during the roll-out of nOPV2.

## Figures and Tables

**Table 1:** Use of OPV2 and subsequent emergences first detected in Africa or elsewhere (1 May 2016 to 1 August 2024).

	Country ^i^	Vaccine Doses (Million) ^ii^		cVDPV2 Emergences ^iii^	
		mOPV2 or tOPV	nOPV2	Derived from mOPV2 or tOPV	Derived from nOPV2
Africa		483	1170	59	18
	Nigeria	163	657	10	2
	DRC	53	89	17	5
Elsewhere		196	20	21	0
Total		679	1190	80	18

^i^ Select countries of interest are shown. ^ii^ Data: POLIS campaigns marked completed with start dates between 1 May 2016 and 1 August 2024. ^iii^ Emergences first detected in location with mean estimated seeding date after 1 May 2016.

## Data Availability

The code is available here: https://github.com/peakcm/polio.
